# The Usefulness of Bioelectrical Impedance as a Marker of Fluid Status in Patients With Congestive Heart Failure: A Systematic Review and Meta-Analysis

**DOI:** 10.7759/cureus.37377

**Published:** 2023-04-10

**Authors:** Daniel Hanna, Iftekhar Baig, Robert Subbiondo, Umair Iqbal

**Affiliations:** 1 Internal Medicine, Hospital Corporation of America (HCA) Blake Florida, Bradenton, USA; 2 Cardiology, Hospital Corporation of America (HCA) Blake Florida, Bradenton, USA; 3 Internal Medicine, Geisinger Commonwealth School of Medicine, Danville, USA

**Keywords:** bioelectrical impedance, biomarker, extracellular water, total body water, heart failure

## Abstract

Bioelectrical impedance analysis (BIA) is a method that measures electrical currents conducted through water, which assesses fluid status by measuring extracellular water (ECW), total body water (TBW), and resistance (R). Limited studies are done to evaluate the utility of BIA in patients with congestive heart failure (CHF), and therefore, we performed a systematic review and meta-analysis to evaluate this. A comprehensive literature search was performed at Medline and Embase until March 2022. Our primary outcome was a comparison of TBW and ECW between patients with CHF and controls. Our secondary outcome was to compare R between the groups. All analysis was conducted using RevMan 5.4 software. Six studies with 1,046 patients met our inclusion criteria. Out of 1,046 patients, 526 had CHF and 538 had no CHF. Among patients with CHF, all 526 had decompensated CHF. There was no significant difference in TBW between patients with heart failure and the control group (mean deviation (MD) = 1.42 (-0.44-3.27), percent of variation (I2) = 0%, p = 0.13). ECW was significantly higher with an assessment of BIA in heart failure patients compared to patients in the control group (MD = 1.62 (0.82-2.42), I2 = 0%, p < 0.0001). Resistance of extracellular fluid was significantly lower in the heart failure group (MD = -45.64 (-72.88--18.41), I2 = 83%, p = 0.001). Publication bias was deferred as the number of included studies was less than 10. BIA can be helpful in ambulatory and inpatient setting to identify patients’ fluid status, which can improve outcomes. However, larger prospective studies are needed to further evaluate the usefulness of BIA in the CHF population.

## Introduction and background

Congestive heart failure (CHF) is currently the leading cause of hospitalizations across the United States and Europe, affecting around 26 million patients. The worldwide financial burden is $108 billion per year [1]. The mortality rate for hospitalized patients with CHF in the USA was found to be around 10% at 30 days, 25% at one year, and 45%-60% over five years of follow-up [2], while the lifetime risk of heart failure is found to be 33% for males and 28% for females ages 55 and up [3].

Traditional monitoring for heart failure exacerbations including patients’ fluid status is often a clinical finding, such as daily body weight, oxygen demand, lower extremity edema, or pulmonary crackles upon auscultation. However, before patients present with these signs and symptoms, they may have asymptomatic structural or functional cardiac abnormalities that serve as precursors of CHF [4-6]. Therefore, the ability to manage and measure fluid retention can be substantial for the treatment of CHF. Currently, there are only invasive methods when monitoring fluid and volume status. Examples of such devices are CardioMEMS and OptiVol, which only measure intrathoracic impedance. While brain natriuretic peptide (BNP) testing, chest radiography, and cardiac ultrasonography are currently being used, they fail to adequately address fluid retention.

Bioelectrical impedance analysis (BIA) is a fast, safe, and noninvasive method that uses the body’s alteration of electrical current through water and tissue at different frequencies through cell membranes to acquire different impedances to assess fluid retention. BIA has been introduced in the medical fields of nephrology, hepatology, nutrition, and rehabilitation [7-9]. Body composition is generally divided into fat-free mass (FFM) and fat mass. FFM contains bone minerals and total body water (TBW), which is the total of intracellular water (ICW) and extracellular water (ECW), which makes up 44% and 29% of body weight in euvolemic humans [10]. Typically, patients with CHF have a loss of muscle mass and function compared to healthy individuals. Since muscles contain a higher percentage of water, which in turn results in less resistance (R), there should be a higher resistance, TBW, and ECW seen in patients with CHF [10].

The BIA test involves placing two electrodes on the person’s right hand and foot, and a low-level electrical current is sent through the body. These electrical currents are usually conducted through water due to their low resistivity [11]. Impedance values are then used to quantify ECW and TBW. Resistance (R), measured in ohms, is the opposition of the tissue to the flow of electrons, which is also measured by the application of these alternating currents when applied to ECW and ICW [12]. BIA can provide estimates of body water in select patients who may present with acute heart failure.

In this manuscript, we sought to systemically review the literature and study the impact of BIA in regard to a patient’s fluid status in the acute heart failure setting.

This abstract was previously presented at the Florida Chapter of the American College of Cardiology (FACC) Annual Meeting Abstract and Case Presentation in Orlando, FL, USA, on August 21, 2022.

## Review

Results

**Figure 1:**
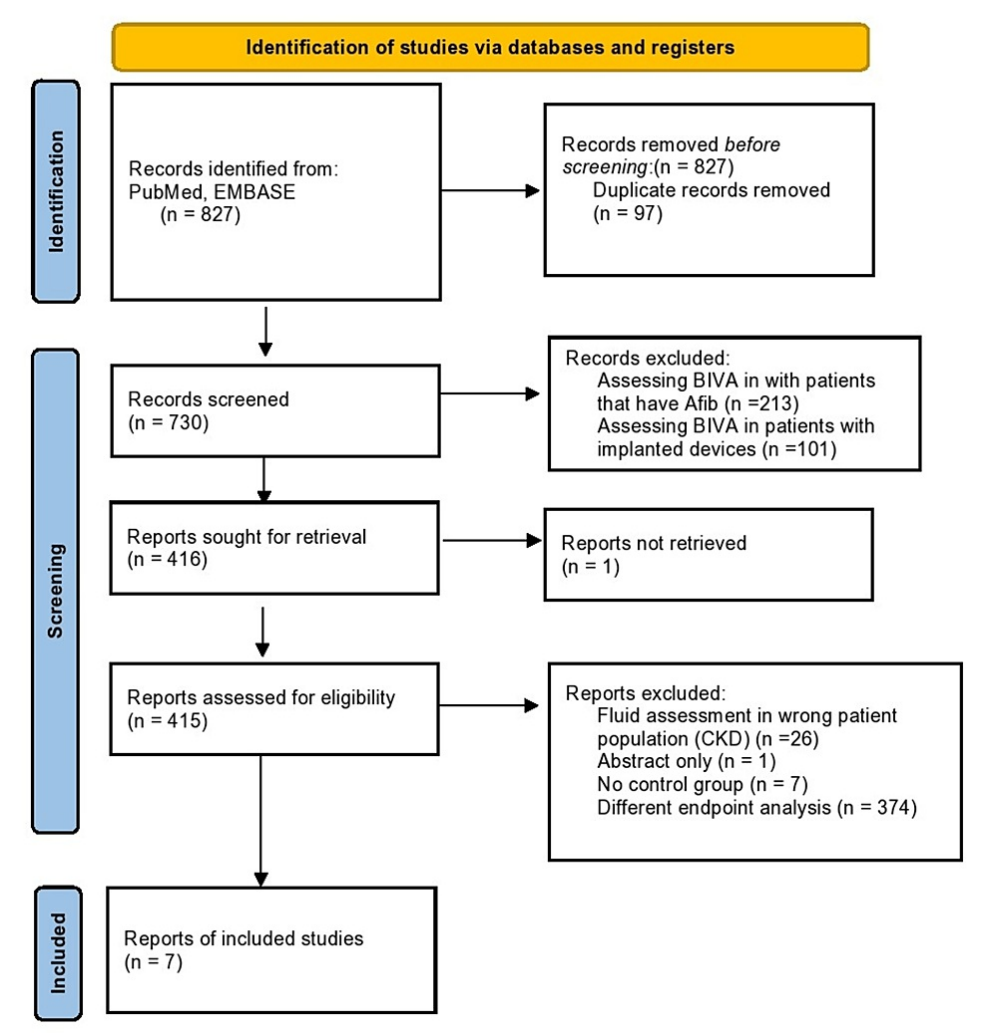
PRISMA flowchart PRISMA: Preferred Reporting Items for Systematic Reviews and Meta-Analyses, BIVA: bioelectrical impedance vector analysis, CKD: chronic kidney disease

**Figure 2:**
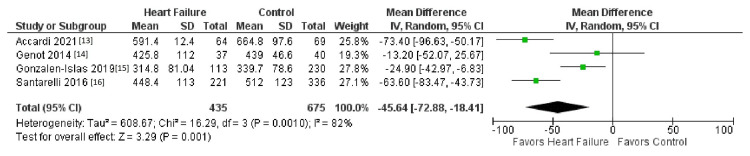
Comparison of the outcome of resistance SD: standard deviation, CI: confidence interval

**Figure 3:**
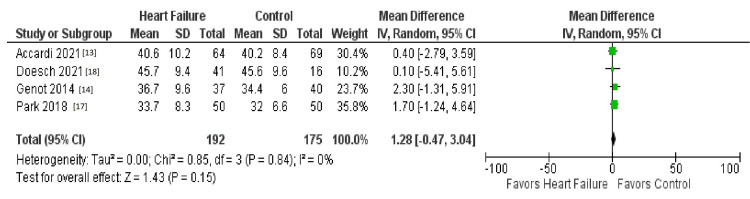
Comparison of the outcome of TBW TBW: total body water, SD: standard deviation, CI: confidence interval

**Figure 4:**
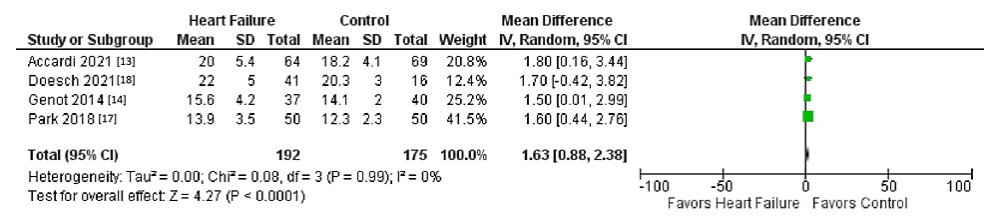
Comparison of the outcome of ECW ECW: extracellular water, SD: standard deviation, CI: confidence interval

The initial search strategy revealed a total of 827 studies. After the removal of duplicates, 730 underwent title review, excluding 314 records. Of the remaining 416 studies, 397 were excluded due to a lack of reporting outcomes of interests and insufficient data. The remaining 13 studies underwent full-text review and yielded a final article count of six. Figure 1 further elaborates on our systemic literature search for our study using the Preferred Reporting Items for Systematic Reviews and Meta-Analyses (PRISMA) analysis. Six studies with a total of 1,046 patients met our inclusion criteria. Using the Newcastle-Ottawa quality assessment form, we found that five out of the six studies were found to be good quality, and one was fair quality. The acute heart failure group included 526 patients, while the non-heart failure group included 538 patients (Table 1 and Table 2). Table 1 and Table 2 further show the demographics and information of all the studies, such as gender, age, and body mass index (BMI). There was no difference in TBW between patients with heart failure and the control group (mean deviation (MD) = 1.42 (-0.44-3.27), percent of variation (I2) = 0%, p = 0.13) (Figures 2-4). ECW was significantly higher with the assessment of BIA in heart failure patients compared to patients in the control group (MD = 1.62 (0.82- 2.42), I2 = 0%, p = 0.0001) (Figure 4). R was significantly lower in the heart failure group (MD = -45.64 (-72.88- -18.41), I2 = 83%, p = 0.001) (Figure 1). Publication bias was deferred as the number of included studies was less than 10. Figure 1 illustrates the PRISMA analysis.

Figure 2 shows a comparison of the outcome of resistance [13-16]. Figure 3 shows a comparison of the outcome of TBW [13,14,17,18]. Figure 4 shows a comparison of the outcome of ECW [13,14,17,18]. Table 1 and Table 2 show our baseline characteristics of the included studies.

**Table 1:** Baseline characteristics of the included studies HF: heart failure, NHF: no heart failure

Study, year	Accardi, 2021 [13]	Génot, 2015 [14]	Park, 2017 [17]	Doesch, 2012 [18]	González-Islas, 2019 [15]	Santarelli, 2016 [16]
Sample size, number	133	77	100	57	343	336
HF	64	37	50	41	113	221
NHF	69	40	50	16	230	115
Study design	Prospective	Prospective	Prospective	Prospective	Prospective	Prospective

**Table 2:** Baseline characteristics of the included studies (continuation) HF: heart failure, NHF: no heart failure, BMI: body mass index, N/A: not applicable

Baseline demographics							
Study, year		Accardi, 2021 [13]	Génot, 2015 [14]	Park, 2017 [17]	Doesch, 2012 [18]	González-Islas, 2019 [15]	Santarelli, 2016 [16]
HF	Mean age	69.3 ± 15.0	71.5 ± 13.6	70.2 ± 11.3	63 ± 12	56.7 ± 17.4	79 ± 8
	Gender (male)	56%	54%	50%	88%	50.4%	45%
	BMI	29.5 ± 6.1	N/A	24.2 ± 4.1	27 ± 4	31.4 (25.3-35.3)	N/A
NHF	Mean age	52.5 ± 11.2	64 ± 14.5	66.1 ± 11.6	61 ± 11	55.9 ± 17.1	76 ± 12
	Gender (male)	46%	45%	52%	75%	46%	53%
	BMI	25.9 ± 4.0	N/A	24.6 ± 3.4	28 ± 5	27.9 (25.3 - 35.3)	N/A
Hospitalized		No	Yes	Yes	Yes	No	Yes
Outpatient		Yes	No	No	No	Yes	No

Discussion

Strategic monitoring and novel markers to guide CHF management remain to be an area of interest and of great importance [19,20]. There are no current concrete guidelines in the assessment of volume and fluid status when assessing acute heart failure patients. The current American College of Cardiology guidelines are only for making the diagnosis of acute heart failure, and even then, there is only one class 1 level A recommendation, which is the measurement of BNP (class 1 level A), while the use of echocardiography, chest X-ray, electrocardiograms, troponin marker, creatine, and electrolytes (sodium and potassium) all fall in class 1 level C [21]. In 2015, the European Society of Cardiology published a paper proposing a multidisciplinary algorithm for acute heart failure patients’ diagnosis and care [22]. One of the proposed steps was the assessment of intravascular volume status. Our goal in this paper is to introduce a unique approach to monitoring heart failure by evaluating a patient’s fluid and volume status.

The main composite of the human body is water, measured as TBW, which is comprised of ICW (55%-65%) and ECW (35%-45%) [23]. The management of these percentages is by water and sodium metabolism, which can be modified and distorted in several different pathologies, such as CHF [24]. As a result of these metabolic changes, patients exhibit typical signs of CHF such as dyspnea, jugular venous distention, and increased cardiac filling pressures, which causes ventricular overloads triggering the synthesis of BNP [25]. Currently, to accurately measure a patient’s fluid and pressure status, patients would have to undergo a procedure that involves the placement of an implanted pulmonary artery pressure or left ventricular pressure monitoring system, an invasive procedure that accrues risk and increased cost to the patient and to the hospital system. There is a subset of heart failure patients that do have such measurement in which they have something called a CardioMEMS device, which is a permanently implantable device that is placed in the distal pulmonary artery to try and accurately measure changes in pulmonary artery pressure. Such changes are thought to be a surrogate for fluid retention in the lungs in patients with heart failure. While the CardioMEMS device possesses great potential regarding fluid status in decompensated heart failure, patients must be eligible and willing to undergo the procedure. Additionally, there is an added cost to the patient, which some may not be able to afford. Furthermore, patients who do not qualify for this will not be able to have their fluid status monitored, whereas BIA can be used in all patients and all settings without needing to satisfy any criteria [26].

There has been another meta-analysis that did assess impedance monitoring in patients with heart failure, specifically with implanted devices such as CardioMEMS, OptiVol, and CorVue [27]. These devices, however, do not measure TBW, ECW, or R and instead measure left atrial pressures, pulmonary artery pressures, and right ventricle pressures. However, this meta-analysis did not measure BIA in their study and instead measured heart failure-related admissions rate, all-cause mortality, and combined heart failure-related admissions rate and all-cause mortality. Their study showed no difference in heart failure-related admissions rate, all-cause mortality, or combined admission rate and all-cause mortality.

To the best of our literature search, this is the first medical review and meta-analysis that comprehensively assesses the use of external BIA as a marker of acute heart failure exacerbations in the inpatient or outpatient setting. Some studies revealed that patients who present to the hospital in an acute heart failure exacerbation have a higher TBW and ECW with an associated higher resistance. Génot et al. [14] did not find a statistical or clinical significance when looking at ECW, TBW, or even resistance between heart failure patients versus controls. Accardi et al. [13], however, found a difference in ECW and TBW values that were significantly higher in CHF patients as compared to adults without CHF, while Park et al. [17] also found that ECW was higher in the CHF population. Doesch et al. [18] did not find any difference in TBW or ECW. Regarding resistance, Génot et al. [14] did not find a statistically significant difference when comparing CHF patients to the control group. Accardi et al. [13] did not comment on the resistance throughout the paper; however, values that were captured and included in the paper showed a difference between the control group and heart failure patients. González-Islas et al. [15] and Santarelli et al. [16] showed that there was a difference in resistance between the control and acute heart failure groups.

Overall, there was no significant difference in TBW when comparing CHF patients to the control. However, when looking at ECW, there was a substantial difference between the two groups. We found that CHF patients had a higher ECW content when compared to the control group. Theoretically, we would expect to see TBW also higher in the CHF group. It is theorized that patients with CHF have an impaired ability to compensate due to electrolyte abnormalities, specifically potassium and sodium [28]. Normally, a healthy person would have the ability to compensate for an increase in extracellular water by shifting excess water to the intracellular compartment. This could explain why the control group showed no major difference in TBW when compared to CHF as these patients had the ability to compensate.

Interestingly enough, there were two other markers that previous papers used to assess volume status in acute heart failure patients. Due to the lack of reporting data in the included studies, we were unable to meta-analytically evaluate the utility of these markers. Both hydration index and phase angle were used as markers in heart failure patients [15,16,29]. The hydration index is a value that can estimate body hydration as well as the percentage of fat-free mass using resistance and reactance. The phase angle is a measurement of the integrity and health of the cell membrane. The phospholipid bilayer in the cell membrane acts as a capacitor as electrons are stored in the membrane. The phase angle has the ability to pick up and measure the stored electrons. If the cell membrane is damaged or if there is increased ECW, the phase angle will be measured low [30]. If more studies were to be published using these markers, there could possibly be a more robust method for managing patients with acute heart failure exacerbations.

## Conclusions

Linking BIA with molecular digital makers could help give physicians important information regarding volume and fluid status to help with medical decision-making for patients with CHF. With a difference of ECW and R in heart failure patients compared to the control, external BIA could possibly be used in the future in the emergency room, inpatient, or clinical environment to assess for appropriate diuresis and possibly catch patients before they start exhibiting symptoms of heart failure and requiring hospitalization. Future larger prospective studies are needed to further evaluate the significance of BIA in patients with CHF.
